# Bayesian Growth Modeling and Length-Based Indicators: Stock Assessment of Nile Tilapia (*Oreochromis niloticus*) in Lake Nasser, Egypt

**DOI:** 10.3390/biology15110868

**Published:** 2026-05-31

**Authors:** Manar Abdellatif, Richard Kindong, Khaled Y. AbouelFadl, Siquan Tian

**Affiliations:** 1 College of Marine Living Resource Sciences and Management, Shanghai Ocean University, Shanghai 201306, China; manar201569@gmail.com; 2 National Engineering Research Centre for Oceanic Fisheries, Shanghai 201306, China; 3 Key Laboratory of Sustainable Exploitation of Oceanic Fisheries Resources, Ministry of Education, Shanghai 201306, China; 4 National Data Centre for Distant-Water Fisheries of China, Shanghai 201306, China; 5 Key Laboratory of Oceanic Fisheries Exploration, Ministry of Agriculture and Rural Affairs, Shanghai 201306, China; 6 Faculty of Fish and Fisheries Technology, Aswan University, Aswan 81628, Egypt

**Keywords:** stock assessment, data-limited fisheries, Bayesian inference, fish ageing, length-based indicators, Nile tilapia

## Abstract

The present study incorporates age- and length-based methods to estimate the stock status of Nile tilapia (*Oreochromis niloticus*) in the inland fisheries of Lake Nasser, Egypt. In 2021, a total of 570 specimens were sampled to determine the length–weight relationships, condition factor, maturity schedules, age structure and growth parameters. The age estimations were quite accurate, suggesting a lifespan of 5 years. Females attained 50% maturity at a length of 27.85 cm and age of 2.30 years, while males attained maturity at 29.93 cm and 2.45 years. The von Bertalanffy model best described growth parameters using the Bayesian approach. Length-based reference points produced from aLBI indicate a close association between length at initial maturity (L_mat_ = 29.3 cm) and optimal harvest length (L_opt_ = 30.8 cm). The Froese indicators indicate that all the proportions of mature fish (P_mat_ = 44.8%), fish of optimum size (P_opt_ = 35.6%), and big spawners (P_mega_ = 18.1%) were below sustainability targets. The results indicate that non-selective fishing gear is responsible for growth and recruitment overfishing by harvesting fish at, or below, maturity. The integrated Bayesian–aLBI approach offers a rigors and transparent assessment for inland fisheries with limited data and support for size-based management actions to rebuild the Nile tilapia stock in Lake Nasser.

## Simple Summary

Nile tilapia is a major species in Lake Nasser and a significant source of food and income in Egypt. However, its stock status remains poorly assessed because of limited biological data. In this study, we combined age validation, growth estimation, maturity analysis, and length-based indicators to assess the status of the fish population. The results showed a negative allometric growth pattern, maturity at around 2 to 2.5 years, and a lifespan of up to 5 years. Many fish are caught before they reach maturity, and the catch lacks enough mega-spawner individuals, as indicated by length-based indicators. These findings suggest that the stock is overexploited and faces severe fishing pressure. Our integrated age- and length-based approach provides a robust stock status framework for helping fisheries managers and supporting their actions to rebuild the stock fishery and ensure long-term sustainability.

## 1. Introduction

Inland capture fisheries are critical for food security, livelihoods, and local economies worldwide; however, their management is often restricted by limited biological data, poor monitoring, and lack of long-term catch and effort series data, particularly in tropical and developing countries [1]. Inland and small-scale fisheries throughout Africa, Asia, and Latin America frequently lack long, standardized time-series of catch and effort, age-structured data, and regular monitoring, making it difficult to apply conventional stock assessment tools and the implementation of sustainable management strategies. In such contexts, methods that can derive robust stock status information from simple biology and length–frequency data are particularly useful.

Nile tilapia (*Oreochromis niloticus*) is one of the most important species for aquaculture and inland capture fisheries in Africa and plays a primary role in food and nutrition security in Egypt. In Egypt, *O. niloticus* dominates aquaculture and inland fisheries, accounting for 144,007 metric tons (54.51%) of the total lake production and 21,047 metric tons (73.23%) of the annual catch of Lake Nasser, despite declining landings over the last three decades, particularly between 2010 and 2018 [2]. Lake Nasser, Egypt’s largest man-made lake and a major source of animal protein in the south, covers an area of 5248 km^2^ and supports multi-gear motorized fishing using gillnets and trammel nets that exploit a wide variety of tilapia size classes [3,4,5]. Inland fisheries have more challenges due to their low priority and limited government support. Although previous studies have described aspects of tilapia biology, growth, and condition in Egyptian inland waters, there is still a lack of integrated evidence on how age structure, growth dynamics, maturity patterns, and length-based exploitation indicators together describe the current stock status of Nile tilapia in Lake Nasser.

Accurate estimates of key life-history parameters are required for the sustainable management of the Lake Nasser tilapia fishery; however, previous studies have reported substantial variability in maturity, longevity, and growth across Egyptian inland systems. Tilapia is an omnivore with broad-range ecological tolerance, rapid growth, disease resistance, and high reproductive capability, which enhances its value in both aquaculture and natural fisheries [6,7,8,9,10]. Fish in Lake Nasser have also been observed to have high condition factors (>2); there are some large-bodied individuals, likely reflecting favorable environmental conditions such as food availability, suitable water quality, and a stable habitat that can support good growth and large individuals [11]. However, the low proportions of mature fish and mega-spawners indicate recruitment overfishing, while an insufficient number of large fish individuals indicates growth overfishing (size-selective overfishing); these patterns suggest that gear selectivity and fishing pressure are major factors driving the current population structure.

Length-based methods for assessing stock status assessment methods provide a practical and robust evaluation framework for fisheries, specifically for data-limited fisheries [12,13,14,15,16]. The abundance length-based indicators (aLBI) approach estimates stock status depending on length-based indicators; this approach requires accurate growth and maturity inputs and applies bootstrap uncertainty analyses for these indicators. These indicators include the proportion of mature fish in the population (P_mat_); the proportion of fish that have an optimal size in the population (P_opt_); and the significant size of spawner fish (P_mega_), as produced by [12]. This approach depends on key biological reference points, including asymptotic length (L_∞_), length at first maturity (L_mat_), maximum observed length (L_max_), and optimal length for harvest (L_opt_) [17].

A recent study indicated that Nile tilapia stock in Lake Nasser faces growth and recruitment overfishing; however, it has a relatively high condition factor. The majority of fish smaller than the mature length (L_mat_) and the use of multiple fishing gears that capture a wide range of sizes are likely to reduce spawning success and truncate the age structure of the population [3]. Previous studies reported that the Nile tilapia stock in Lake Nasser is overfished [3,18], which emphasizes the urgent need for monitoring and management strategies to help the stock to recover. Although age-based validation and length-based indicator approaches are robust and quick tools for estimating stock status, they are still underused in Egyptian inland fisheries [19]. This limits the development of appropriate management strategies with which to conserve the overexploited tilapia stocks in Lake Nasser.

Therefore, the present study aimed to evaluate the stock status of Nile tilapia in Lake Nasser using an integrated framework that incorporates age validation, growth modeling, and length-based indicators. The study included: (1) estimating the total length–weight relationship, condition factors, length and age at first maturity (L_50_ and A_50_), and age using otolith validation; (2) using multi-model inference and Bayesian von Bertalanffy to estimate growth parameters; and (3) using an aLBI method to estimate length reference points (L∞, L_max_, L_mat_, and L_opt_) and Froese indicators (P_mat_, P_opt_, and P_mega_) to evaluate stock sustainability and evaluate stock status using length-based indicators. This comprehensive approach provides a transparent and scientifically informed framework for assessing data-limited inland fisheries, which can help managers to develop management strategies to ensure stock sustainability.

## 2. Methods

### 2.1. Study Area

**Figure 1:**
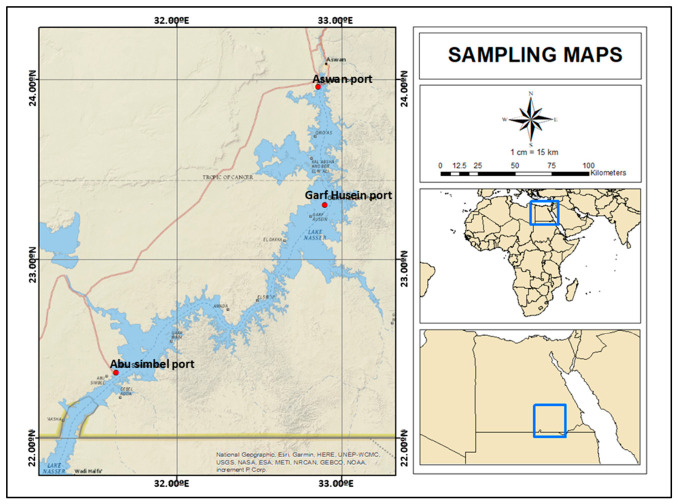
Map of Lake Nasser, Egypt. Red dots correspond to the landing sampled sites in the study.

Lake Nasser (Figure 1) is the northern portion of Egypt’s Aswan High Dam, which was built in the 1960s for storing the River Nile’s floodwater. It is located between latitudes 21.8 °N and 24.0 °N, and longitudes 31.3 °E and 33.1 °E. The area covers about 5248 km^2^, with an overall capacity of 165 km^3^, and an average depth of 130 m [20]. The lake is around 550 km long (more than 350 km in Egypt and the remainder in Sudan) and 35 km wide at its broadest point [21].

### 2.2. Sample Collection

A total of 570 specimens were collected randomly from different boats every month except November during 2021 from commercial ports of Lake Nasser (Aswan in the north, Garf Hussein in the center, and Abu Simbel in the south). The specimens were caught using a trammel net (duk) consisting of three layers, two outer layers with large mesh sizes, and one inner layer with a smaller mesh size. “Ghazel Shabar”, with a mesh size of less than 110 mm, targeted small-sized species (less than 500 g in weight and 25 cm long) and “Ghazel Bolti”, with a mesh size of over 110 mm, targeted large-sized species (more than 500 g in weight and 25 cm long) [4,5]. Most of the hauling boats are motorized, with engines ranging from 9.9 to 25 hp, while a few still use paddles; these boats have a length of 4.5 to 9 m and a width of 1 to 3 m [22,23].

The specimens were collected from the lake’s three ports combined for further analysis. The total length and total weight data were gathered for analysis and measured (cm for total length and g for total weight) and sectioned for macroscopic examination to determine fish sex. The length–frequency distribution of specimens for each month is shown in Table 1.

### 2.3. Length–Weight Relationship

The length–weight relationship parameters estimated using Equation (1) [24]:

(1) $$W=aL^{b}$$

W is the entire weight of the fish, a is the intercept, L is the overall length of the fish, and b is the relationship’s exponent. The growth pattern was classified as b = 3 for isometric growth, b > 3 for positive allometric growth, and b < 3 for negative allometric growth. We used the student’s *t*-test to evaluate significant differences between the b values for males, females and individuals of unidentified sexes compared to the hypothetical value (b = 3). The linear regression analysis using log_10_-transformed linear regression gave the correlation coefficient (r^2^), which represents the strength of the length–weight relationship: R = r^2^.

W is calculated from the logarithm (base 10) in Equation (2):

(2) logW=loga+b.logL

### 2.4. Condition Factor

The Fulton’s condition factor is estimated using Equation (3) [25]:

(3) $$Kc=100\times \frac{W}{L^{3}}$$

where W is the total weight and L is the total length.

### 2.5. Growth Estimation and Maturity Analysis

#### 2.5.1. Gonadosomatic Index

The gonadosomatic index (GSI) is an indirect method of predicting a species’ spawning season and identifying the peak phase of ovarian development. It is estimated using Equation (4):

(4) $$GSI=\left(\frac{gonad\;weight}{fish\;weight}\right)\;\times \;100$$

#### 2.5.2. Maturity Analyses

All specimens were sectioned for determining the sex and gonad maturity stages. The gonads of *O. niloticus* were described according to the scale of Gunderson, with some modifications, into the six stages shown in Table S1 [26].

After that, the data of maturity stage was transformed to a dual maturity classification that indicates immature = 0 and mature = 1 for statistical analysis. Quasibinomial (binned) model was used to bin the data by age class and determine the proportion mature in each age class.

Length at 50% and 95% maturity (L50 and L95) for males and females were estimated using binomial logistic regression from the AquaticLifeHistory package (4.5.1) [27], applying a generalized linear model (GLM) with binomial error structure and logit-link function in the ‘R’ programming environment, version 4.5.1 [28].

Length at maturity for both males and females were estimated using Equation (5) [29,30]:

(5) $$p\left(l\right)\;=\;p_{max}{\left(1+e^{-\ln\left(19\right)\left(\frac{1\;-\;l50}{l95\;-\;l50}\right)}\right)}^{-1}$$

where P(l) is the percentage of the population that has achieved TL and Pmax is the highest percentage of adults. The same GLM structure model was applied using age data to estimate age at maturity (A50 and A95).

#### 2.5.3. Otolith Handling and Growth Estimation

**Figure 2:**
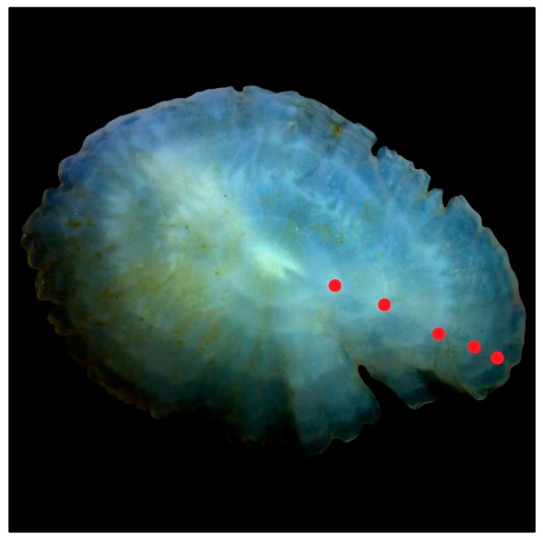
Otoliths of 5 years old female *O. niloticus* from Lake Nasser. Red dots indicating the five annual growth rings used for age determination.

Otoliths were utilized to determine age; both sagittas were removed from 562 specimens. After that, the otoliths were rinsed in water to get rid of any excess tissue, dried, labelled, and stored in plastic vials. We utilized a camera (ABBOT DEC 2000, Carl Zeiss Microscopy GmbH, Göttingen, Germany) attached to a binocular stereomicroscope (ZEISS Stemi 508, Carl Zeiss Microscopy GmbH, Göttingen, Germany) to take digital pictures of each pair of otoliths. The otoliths were immersed in 50% glycerol and lit up with oblique reflected light. The clearest picture was chosen for analysis [31]. Two types of rings were identified: an opaque white ring representing summer and a translucent ring that appeared black, representing winter (Figure 2).

The age was calculated by counting the pairs of clear and translucent growth rings. The otolith sections were examined independently by three researchers. The accurate age was found by agreeing on two or three measurements. The average percent error (APE) was used to assess precision and bias using Equation (6) [32]:

(6) $${APE}_{j}=100\%\times \frac{1}{R}\sum_{j=1}^{R}\frac{\left|X_{ij}-X_{j}\right|}{X_{j}}$$

APE_j_ is the average percent error for the jth sample; x_ij_ is the ith age estimate for the jth sample; x_j_ is the mean age estimate for the jth fish; and R is the frequency of ageing for each sample.

When the mean is calculated for a large number of fish, it represents the average error rate. Chang proposed a more robust estimate based on mean absolute deviations and replaced standard deviation [33]. The method using the coefficient of variation to measure the average percent error (APE) is shown in Equation (7):

(7) $${CV}_{j}=100\%\times \frac{\sqrt{\sum_{i=1}^{R}\frac{{\left(X_{ij\;}-\;X_{j}\right)}^{2}}{R\;-\;1}}}{X_{j}}$$

CV_j_ represents the age precision estimate specifically for the jth fish. Similar to the equation for APE, it can be averaged among fish to yield a mean CV.

We employed a multi-model inference (MMI) to determine the rate at which *O. niloticus* grows, as shown in Table 2.

MMI is a method that compares multiple growth models using the Akaike Information Criterion (AIC) to select the best fit, ensuring the uncertainty for the model [37]. It evaluates how well different models work and selects the one with the lowest AIC (AICmin) value as the best fit [27]. AICc, which corrects AIC for small sample sizes, is estimated using Equation (11):

(11) $${AIC}_{c}=AIC+\frac{2K(K\;+\;1)}{n\;-\;K\;-\;1}$$

AIC is calculated as nlog(σ^2^) + 2k, where k is the total number of parameters plus one for variance σ^2^, and *n* is the sample size. The models were arranged based on the value of Δ. Values from 0 to 2 were thought to have the most support, values from 2 to 10 had less support, and values greater than 10 had the least support [38]. We sorted the models based on their AIC difference (Δ), which was estimated for the three models (i = 1–3), as shown in Equation (12):

(12) $$\Delta i={AIC}_{ci}-{AIC}_{min}$$

The AIC weights were estimated using Equation (13):

(13) $$W_{i}=\frac{e^{\left(-\frac{\Delta i}{2}\right)}}{\sum_{j=1}^{3}\left(e^{\left(\frac{\Delta i}{2}\right)}\right)}$$

#### 2.5.4. Bayesian Inference Model

We applied the Stan programming language and the brms package to fit the von Bertalanffy growth model using Bayesian inference methods and estimate growth parameters [39]. We fixed t_0_ (the theoretical age at a length of zero) and calculated the von Bertalanffy growth parameters using Equation (14):

(14) $${TL}_{i}=L_{\infty }*\left(1-e^{\left(-K_{ti}\right)}\right)$$

where TL_i_ is the total length of fish i, L_∞_ is the average maximum length indicated, K is the growth coefficient, and t_i_ is the length of fish i.

The Bayesian von Bertalanffy growth model was fitted using 4 MCMC chains running for 40,000 iterations, which included 4000 warm-up iterations (no thinning). This resulted in effective sample sizes of >2500 and R = 1.00 for all parameters. The posterior predictive checks were used to evaluate model suitability by comparing observed lengthfrequency distributions with replicated datasets produced from the posterior predictive distribution.

### 2.6. Stock Status Using aLBI

The (aLBI) approach is a data-limited stock assessment method that estimates fish population status using length–frequency data and a significant history parameter, including asymptotic length (L_∞_) and length at first maturity (L_m_ or L_50_). It uses Monte Carlo and bootstrap methods for estimating key biological parameters: asymptotic length (L_∞_), length at first maturity (L_50_ or L_m_), optimum length (L_opt_), and maximum observed length (L_max_). We used the (aLBI) package (4.5.1) [17] to check on the status of the stocks. The package can be obtained from GitHub (for the most recent version, version 2.54.0) or CRAN (https://cran.r-project.org/package=aLBI, accessed on 9 January 2026).

#### 2.6.1. Key Length Parameters

We used the Fishpar function to estimate asymptotic length (L_∞_), highest length (L_max_), optimum length (L_opt_), and maturation length (L_mat_) within the aLBI package.

We used brms outputs (L∞ and L_mat_) as inputs in aLBI. L_mat_ and L_opt_ were obtained using Equation (15) [40]:

(15) $$\;\log\left(L_{mat}\right)=0.8979\left(\log\left(L_{\infty }\right)\right)-0.0782$$

Further, L_opt_ was estimated from L_mat_ using Equation (16):

(16) $$\log\left(L_{opt}\right)=1.053(\left(\log\left(L_{mat}\right)\right)-0.0565$$

These parameters were developed primarily to examine sensitivities in parameter estimations to make sure the model could accurately represent differences in growing and maturation data.

#### 2.6.2. Estimating Length-Based Indicators (LBIs)

Froese (2004) [12] suggested three length-based indicators (LBIs) that can be used to estimate the stock status and indicate exploitation levels. At first, P_mat_ was defined as the proportion of mature fish in the capture that were longer than L_mat_ (sexual maturity length). The P_mat_ was calculated using Equation (17) [12]:

(17) $$P_{mat}=\sum_{L_{mat}}^{L_{max}}Pl$$

where PL denotes the proportion of fish in the catch composition within a length interval of L.

The second indicator is P_opt_, which is defined as the proportion of fish caught at the optimum length for maximum yield and livelihoods (L_opt_). The P_opt_ was calculated using Equation (18):

(18) $$\;P_{opt}=\sum_{0.9L_{opt}}^{1.1L_{opt}}P_{L}$$

The last indicator is P_mega_, defined as the percentage of larger and older fish (mega spawners) in the catch. It is calculated as the percentage of fish that exceed the optimal length by 10%. The P_mega_ was estimated using Equation (19):

(19) $$P_{mega}=\sum_{1.1L_{opt}}^{L_{max}}P_{L}$$

The FishPar function used a non-parametric bootstrap method to measure uncertainty in aLBIs. Length–frequency data were resampled as individual fish observations, generating 5000 bootstrap replicates that preserved the original monthly sample proportions shown in Table 1. Using length ranges from Monte Carlo simulations, P_mat_, P_opt_, and P_mega_ were found for each sample.

We utilized the 2.5th and 97.5th percentiles of the resulting distributions to make 95% confidence intervals, with limits set at 0% and 100% to ensure that they made biological sense. This method used R’s sample and quantile functions to obtain credible estimates of how much the indicators varied. It also worked well with the Monte Carlo method for length parameters [41].

## 3. Results

**Figure 3:**
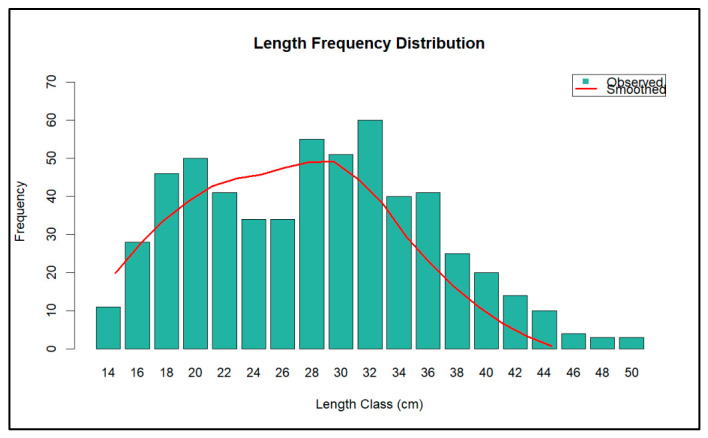
Length–frequency distribution of *O. niloticus*, Lake Nasser.

The histogram of length–frequency distribution is shown in Figure 3. A LOESS (locally estimated scatterplot smoothing) regression was used for the histogram. The smoothed curve (red line) indicates a left-skewed distribution with a modal peak between 26 and 30 cm, followed by a shoulder mode detected between 40 and 44 cm.

### 3.1. Length–Weight Relationship

**Figure 4:**
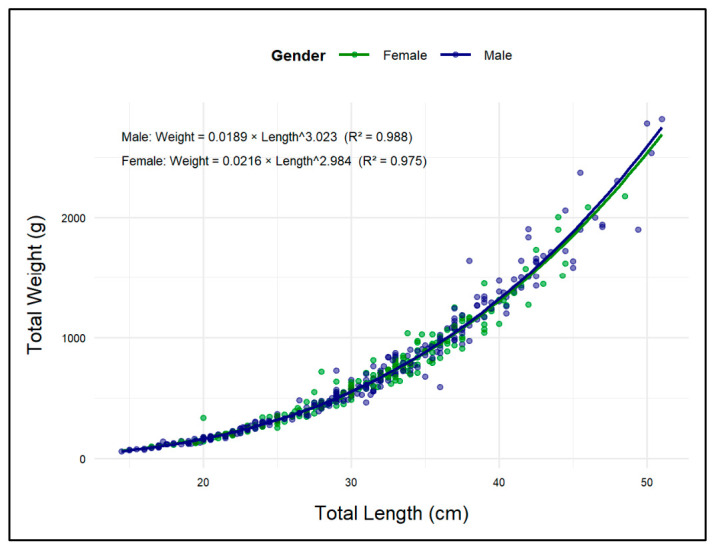
Total length–total body weight relationship of *O. niloticus*, Lake Nasser.

The correlations between total length (TL) and body weight (W) of *O. niloticus* are shown in Figure 4 and represented by the equations W = 0.024TL^3.02^ for males and W = 0.028TL^2.98^ for females. The b value (the exponent of the length–weight relationship) indicates isometric allometric growth for males (b = 3.02, t = 1.137, df = 281, *p* = 0.257) and females (b = 2.98, t = −0.505, df = 223, *p* = 0.614). The fits are good, as indicated by R^2^ in Figure 4.

### 3.2. Condition Factor

**Figure 5:**
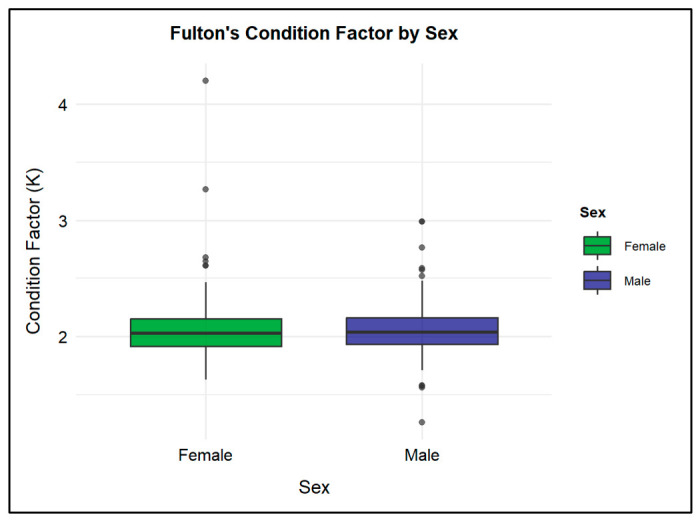
Fulton’s condition factor of *O. niloticus*, Lake Nasser.

The Fulton’s condition factor for males, females, and uncertain-sex individuals ranged from 1.63 to 4.2 and 1.26 to 2.99 for females and males, respectively. All values are more than 1; the results are presented in Table 3 and illustrated in Figure 5.

### 3.3. Growth Estimation and Maturity Analyses

#### 3.3.1. Age Estimation Using Otoliths

**Figure 6:**
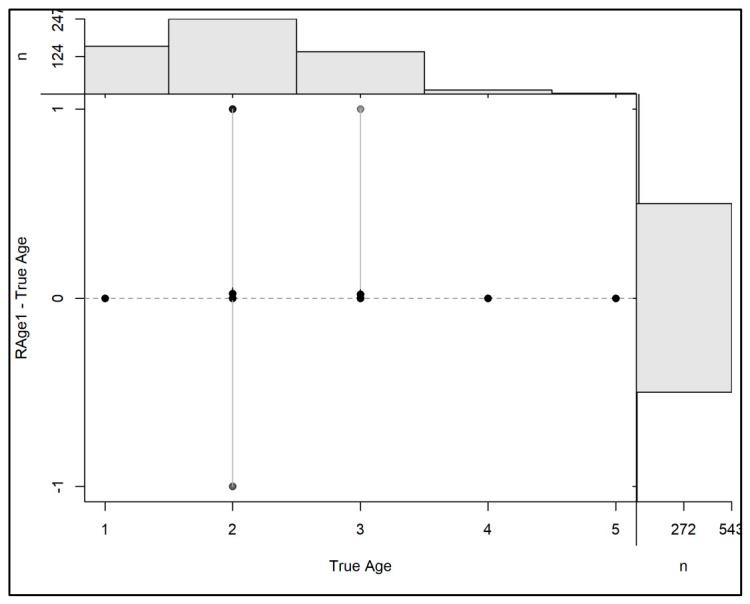
The age-bias plot displays the relationship between the readers-age readings and the true age, including 95% average confidence intervals for *O. niloticus*, Lake Nasser.

The age estimates of *O. niloticus* were highly precise and almost identical among the three readers. Ageing precision for 562 specimens was high between the three readers, with an overall percentage of 92.88%. The mean coefficient of variation (ACV) was 1.85%, and the mean average percent (APE) was 1.42%. The average coefficient of variation (ACV) and average percent error (APE) were also lower than 5%, indicating high precision in age determination (Table 4). The percent agreement was 95.20 (ACV of 3.02%), 94.13 (ACV of 4.80), and 96.44 (ACV of 3.02%) between reader 1 and reader 2, reader 1 and reader 3, and reader 2 and reader 3, respectively (Figure 6).

#### 3.3.2. Gonadosomatic Index

**Figure 7:**
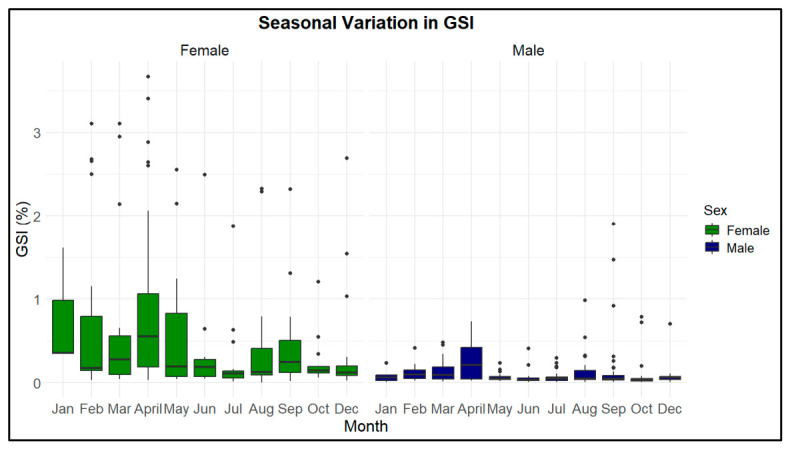
Gonadosomatic index for *O. niloticus*, Lake Nasser.

The monthly fluctuation of the gonadosomatic index (GSI) for both male and female *O. niloticus* is illustrated in Figure 7 and presented in Table S2, indicating that spawning peaks for both sexes occur in April at 3.67 for females and 0.73 for males. GSI values range from 0.03 to 3.67 (average = 0.89) for females and from 0.02 to 0.73 (average = 0.27) for males. Prior to April (from January to March), the GSI for males and females increases, since it represents the preparatory phase preceding the peak in April. Conversely, from May to July, during the post-spawning phase, the GSI declines for both sexes. Between August and September, there is an obvious rise in GSI for both sexes, potentially indicating a minor secondary spawning season. At the end of the year (from October to December), the GSI stays decreased for both sexes, signifying an inactive reproductive phase.

#### 3.3.3. Maturity Analyses 

**Figure 8:**
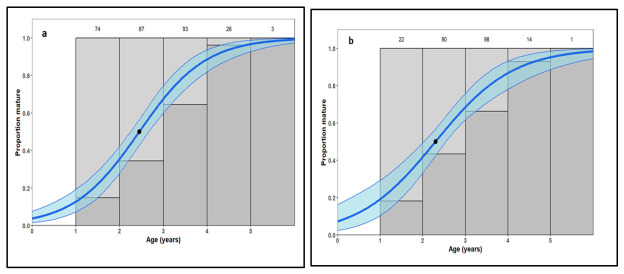

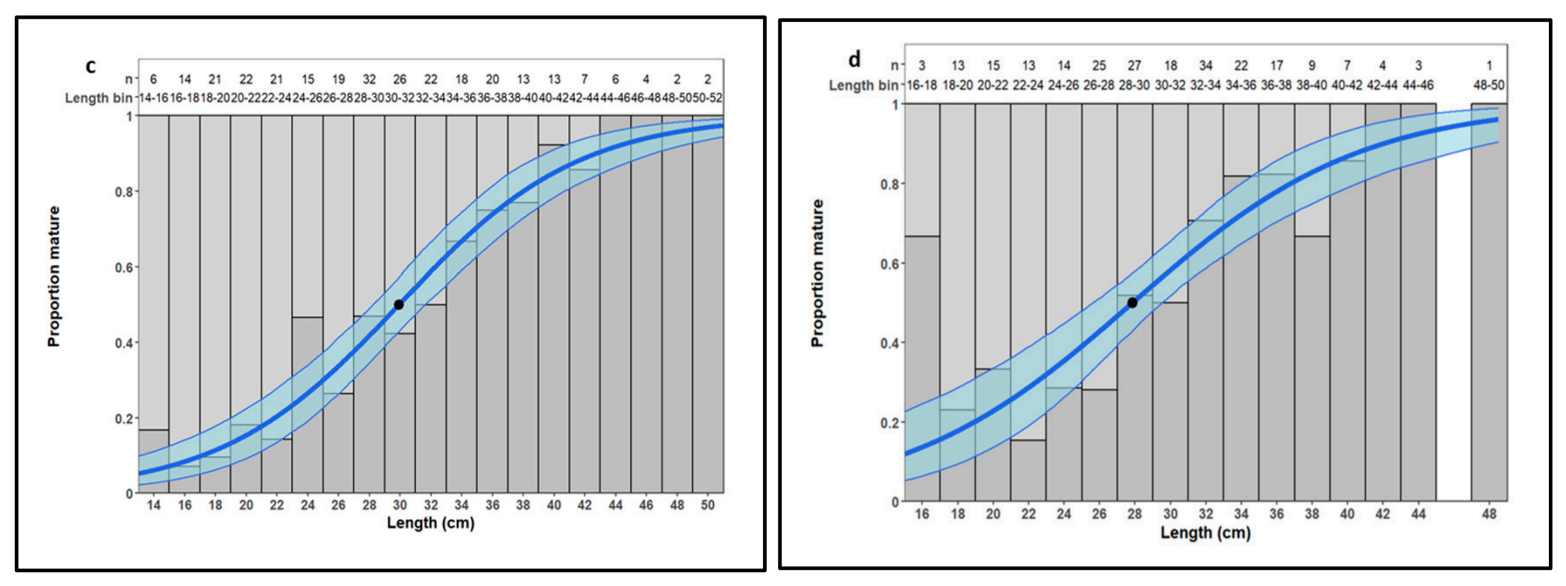
Maturity age and length of *O. niloticus*, Lake Nasser: (**a**) age at maturity for males; (**b**) age at maturity for females; (**c**) length at maturity for males; and (**d**) length at maturity for females.

The maturity analysis results of Quasibinomial model (bin = 2) for male and female *O. niloticus* are almost identical (Table 5 and Figure 8). Females attain maturity at slightly smaller sizes and ages than males, and they mature faster. Females reached 50% maturity at a length (L_50_) of 27.85 cm and age (A_50_) of 2.30 years. Males reached 50% maturity at a later stage, with an L_50_ of 29.93 cm and an A_50_ of 2.45 years. For both sexes, the L_50_ is 29.41 cm and the A_50_ is 2.47 years.

#### 3.3.4. Growth Estimation Using AquaticLifeHistory

**Figure 9:**
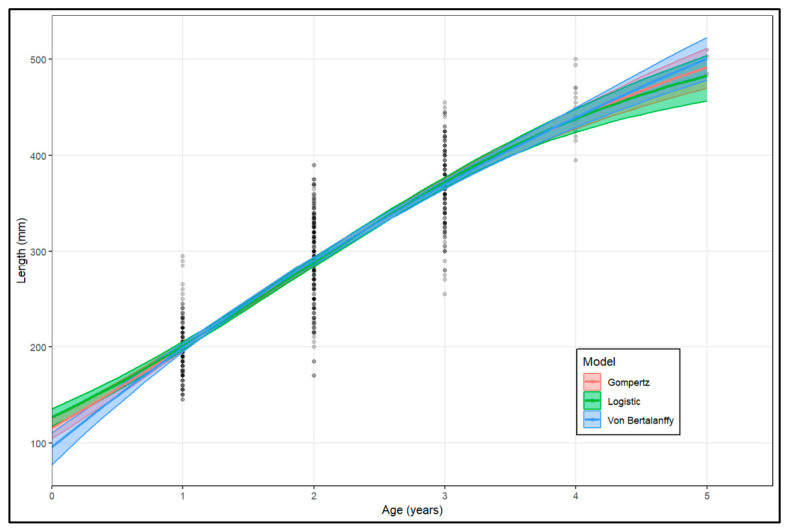
The three growth curves for *O. niloticus*, Lake Nasser.

A total of 562 otoliths were examined to estimate fish age. They ranged in size from 14.5 cm to 51 cm (average = 29 cm), with total weights ranging from 58.3 g to 2814 g (average = 611.92 g). We calculated model-averaged length at age using MMI. Three growth models (von Bertalanffy, Logistic, and Gompertz) were estimated using Estimate_growth(), which is the main function that applies the MMI approach (Figure 9). AIC revealed that the von Bertalanffy model is the best fit, as it has the lowest value (Table 6). Even the residual standard error (RSE) confirmed AIC results between all three growth models and indicated the lowest value for the von Bertalanffy model.

#### 3.3.5. Bayesian LVB, brms (Fixed t0)

**Figure 10:**
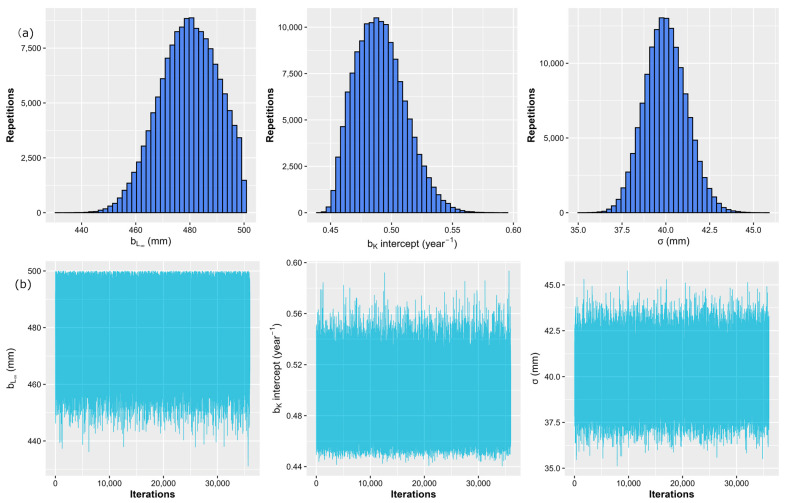
The mixed chains for growth parameters of *O. niloticus*, Lake Nasser: (**a**) unimodal distribution for each parameter (L∞, K, sigma); and (**b**) caterpillar plots for each parameter (L∞, K, sigma).

We used the von Bertalanffy Bayesian inference model. The Gelman–Rubin diagnostic (R^) point estimates are less than 1.2 (=1) for all growth parameters (Table 7), indicating convergence (Figure S1). The chains appear to mix well across all parameters and achieve a stationary posterior (Figure 10) L∞ ranging from 45.57 cm TL to 49.70 cm TL and coefficient of growth (K) ranging from 0.46 to 0.54 year^−1^, using a posterior predictive check that compares observed data to predicted values based on the fitted model. The predicted values from the model are “similar” and closely overlap with the observed data values, indicating that the model fits the data well. The close overlap indicates that the model is suitable for data generation. However, while the model reasonably captures the overall size pattern, the fit is worse, below 200 mm, and does not fully represent the observed bimodal distribution (Figure S2).

### 3.4. Stock Status Using aLBI Model

#### 3.4.1. aLBI Length-Based Reference Points

**Figure 11:**
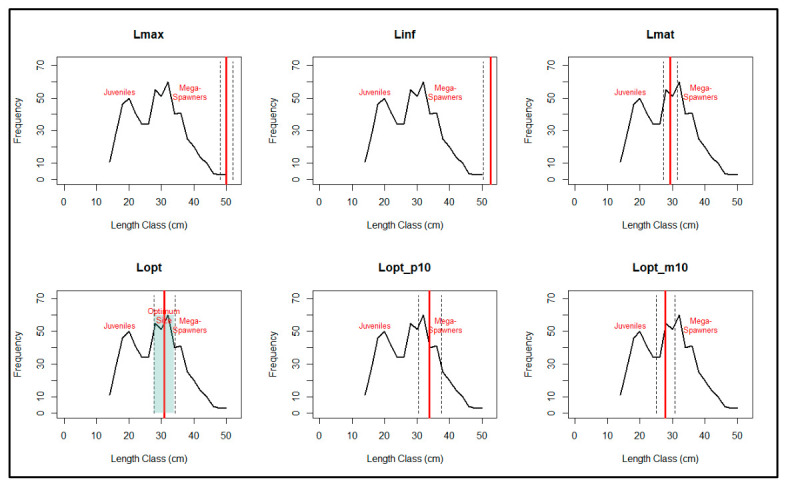
Key length parameters of *O. niloticus* with the frequency line. Solid red lines indicate the mean values, while dotted lines show the upper (Upper_CI) and lower (Lower_CI) confidence intervals.

The aLBI technique calculated multiple length groups for *O. niloticus*, providing extensive information regarding the stock status of the fish. The mean maximum length (L_max_) was 49.98 cm, and the asymptotic length was 52.61 cm, showing that the observed size structure matches expected growth patterns. The estimated average length at first maturity (L_mat_) was 29.32 cm, and the average optimum length (L_opt_) for maximizing yield per recruit while avoiding overfishing was 30.80 cm. L_mat_ and L_opt_ are too close to each other, indicating that the fish stock is overexploited, as the best harvest occurs just after sexual maturity. The mean length of the upper optimal length range is ${L_{opt}}_{+10\%}$, whereas the mean length of the lower optimal length range is ${L_{opt}}_{-10\%}$. These variables (shown in Table 8 and Figure 11) provide a comprehensive overview of the biologically size-based management framework, which will be especially valuable in data-limited assessments.

The length parameter distributions have comparatively narrow ranges, which indicates their robustness, making them suited for size-based stock and management.

#### 3.4.2. Evaluate Stock Sustainability Using the Froese Indicators

**Figure 12:**
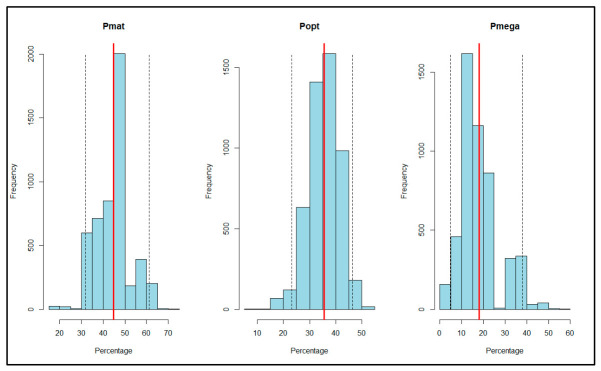
Froese’s length-based indicators frequency of *O. niloticus*. The red line shows the mean values and dotted lines show the upper and lower confidence intervals.

Length-based indicators suggested by Froese (2004) [12] were estimated for *O. niloticus* in Table 9 and Figure 12, which revealed significant variance and were below the target values (100%). The mean percentage of mature fish (P_mat_) was 44.75%, with a CI between 32% and 61.09%, and the mean percentage for optimally sized fish (P_opt_) was 35.56%, with a CI between 23.11% and 46.53%. The average proportion of mega-spawners is 18.14%, which is too close to the target (20%); even the confidence interval (4.82–37.94) is broad and implies considerable uncertainty, indicating that their proportion may not be sufficient to explain the entire range of recruitment variability.

**Figure 13:**
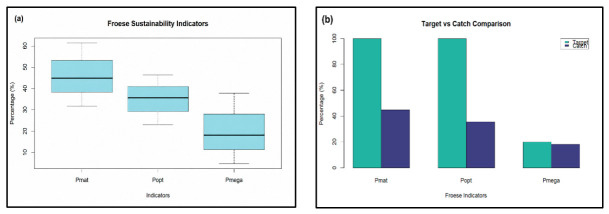
(**a**) Box plot of Froese’s length-based indicators of *O. niloticus* shows the mean, upper, and lower confidence intervals; and (**b**) comparison between Froese target reference point and observed catch proportions for the three length-based indicators of *O. niloticus*.

All three indicators (P_mat_, P_opt_, and P_mega_) are below their sustainability target (Figure 13), revealing that the fish population state is characterized by excessive removal of mature and suboptimal individuals, with only a few large spawners protected.

The growth parameters (L∞, K, and b) and other key length parameters (L_max_, L_mat_, and L_opt_) values reported by multiple authors in various regions are shown in Table 10.

## 4. Discussion

The present study provides an integrated framework for assessing tilapia stock at Lake Nasser, using age validation, growth modeling, maturity analysis and length-based indicators. Our results indicate that the stock is under risk of heavy fishing pressure, with evidence of both growth overfishing and recruitment overfishing. The population still shows a relatively high condition factor and some large-sized individuals; however, the capture structure is dominated by fish near, or below, maturity size, which indicates that the current behavior of exploitation does not allow for enough fish to reach optimal harvest sizes or have a chance to reproduce [12]. It is a historical exploitation approved by previous investigations and is still occurring [3,4].

Length–weight relationship and condition factor studies are critical and important in fisheries science [55]. Length–weight relationships vary regionally and are temporally based on various parameters, including fish size range, environmental conditions, reproductive patterns, food quality and availability, diseases, and competition [56,57,58]. In our study, the length–weight relationship was found to be approximately isometric growth in males and females, showing that isometric growth is still suitable between length and weight. This pattern provides a sign for suitable environmental conditions and enough food availability [35]. The relatively high Fulton condition factor supports that the fish are in a high physical condition [59]. However, adequate body condition does not indicate evidence of low fishing pressure. A population can be heavily harvested while individual fish have high body conditions if the habitat is still productive and has sufficient food sources [60,61]. That is precisely the situation in Lake Nasser, which reflects a productive environment with high fishing pressure.

The maturity results indicate that Nile tilapia in Lake Nasser reach sexual maturity at lengths of around 28 and 30 cm (TL) and ages of about 2.3–2.5 years. Females reach maturity stages slightly earlier and at smaller sizes than males, which is consistent with the life cycle of many teleosts [62,63]. This difference may reflect sex-specific energy attribution to development and reproduction, as well as varying vulnerability to fishing gear. Furthermore, the estimated length at first maturity was close to the estimated optimal harvested length, indicating that many individuals are vulnerable to being caught before, or near to, the maturation development stage. This pattern serves as a classic indicator of recruitment overfishing, as it indicates that few fish are being allowed to reproduce before being harvested [12].

The GSI is a significant indicator that helps us understand how the fish are developing [64,65]. The GSI pattern indicates a spawning peak in April, with the possibility of a second reproductive pulse later in the year. Our results agree with AbouelFadl et al. [52] and Wagaw et al. [66], who reported April as a peak for *O. niloticus* in Lake Nasser and the Geray Reservoir, respectively. This seasonal pattern is useful for fisheries management to identify the period in which the stock is highly reproductive and, therefore, most vulnerable to fishing activity [67]. High fishing mortality during, or just before, the spawning season will significantly impact the stock’s reproduction and decline its sustainability.

Age and growth information are essential for assessing fish population status and enhancing the efficacy of fisheries evaluation and management [19]. Fish age examination is a key component for the fisheries field, as it is used to comprehend growth rates, mortality, recruitment, and all life history parameters [68,69,70]. Using otolith estimation, we observed that the lifespan of *O. niloticus* in Lake Nasser is 5 years, which is lower than the lifespan reported for some other populations (9 years) [71] due to fishing gear selectivity and growth overfishing, which truncate older age classes (P_mega_ = 18.1). Compared to our results, shorter lifespans were reported in the River Nile [44,72], a shallow tropical lake in Mexico [45], and in coastal Mississippi [73]. Longer lifespans were reported in Nozha Hydrodrome, Lake Mariout [47], in the El-Bahr El-Faraouny Canal [74], and in Lake Hayq [75].

Growth modeling revealed that the von Bertalanffy was the best fit; the Bayesian estimates confirmed a moderate growth rate and a finite asymptotic size consistent with the observed length structure. This agreement between the multi-model inference and Bayesian approach enhances confidence in parameter estimates [76]. However, the model fit was weaker at the lower end of the size range and did not fully replicate the bimodal length–frequency distribution, which suggests the fish population may be affected by fishing gear selectivity, cohort overlap, or different recruitment cycles, which are taken into account when interpreting population growth dynamics [77]. The Bayesian framework is a vulnerable approach in that concern because it explicitly quantifies uncertainties in estimating the growth parameters [78].

Froese length-based indicators are an effective tool for assessing the health and sustainability of fish stocks, especially in inland lake fisheries, where there are not many data [12,79]. According to the yield-per-recruit theory, allowing individuals to approach the optimal length (L_opt_) enhances biomass accumulation and fisheries efficiency [77]. In our study, the aLBI results support the stock stress; the estimated L_mat_ and L_opt_ were close, indicating that the fishery is capturing fish sizes that are below, or slightly above, maturity. These indicators were aligned with earlier studies that revealed overexploitation of the Nile tilapia stock at Lake Nasser [3,4]. In the context of sustainable exploitation, a larger proportion of the catch should be harvested at the optimal length and the number of meg-spawner individuals should be higher [12]. The values of P_mat_ and P_opt_ were below the proposed reference values; even P_mega_ was below the target value. Combining these indications revealed that few fish individuals survive to the size required for reproduction and yield recruitment.

Individual health does not necessarily correspond to population sustainability; the disparity between the relatively adequate individual condition indices and the low stock assessment indicators underscores the critical need for management. Fish in productive lakes, such as Lake Nasser, remain in good condition even when they are under fishing pressure. As a result, the condition factor should combine with maturity, age structure, and length-based indicators as a complete approach. The comprehensive approach used in our study is, therefore, more biologically informative.

These findings are generally in agreement with previous studies that reported the overexploitation of Nile tilapia in Lake Nasser and even in other Egyptian inland resources [42,43,44]. The differences in parameter estimates are due to variations in habitat location, fish genetics, environmental condition quality, sampling methods, gear selectivity, and local fishing pressure [54,80]. The integration of age-based and length-based methods and combining them in a single framework is particularly critical in data-limited fisheries, as catch and effort series are incomplete or unavailable [81].

In terms of management perspectives, these findings suggest the urgent need to reduce fishing pressure on immature and small-sized fish. Our recommendations are to increase mesh-size selectivity, protect spawning seasons, and enforce regulations to limit landing size. Therefore, these regulations will improve the stock structure over time and help to rebuild the stock yield [62,77]. Furthermore, management strategies should prioritize conserving a high proportion of large spawners and enabling the fish to spawn at least once before catching them, which is essential for sustaining population recruitment. This would help the restoration of age structure, improve spawning potential, and boost long-term production.

In conclusion, the combined biological evidence indicates that the stock of Nile tilapia in Lake Nasser is overexploited, despite individuals exhibiting good somatic condition. The current Nile tilapia stock in Lake Nasser is considered unsustainable due to high fishing pressure and low proportions of mature and large spawner fish. The integrated Bayesian and aLBI approaches used in this study provide an effective framework for investigating inland fisheries with limited data, which can assist in evidence-based management decisions for Lake Nasser.

## 5. Conclusions

The study provides a comprehensive assessment of the growth dynamics and size-based stock assessment for *O. niloticus* at Lake Nasser. Using validated age estimates and Bayesian von Bertalanffy growth modeling, we obtained accurate age determination and consistent growth parameter estimates, with posterior predictive checks confirming a strong model fit to the observed data. Length-based indicators indicate a truncated size structure, with low proportions of mature fish, optimally sized fish, and mega-spawners, indicating heavy fishing pressure and a high risk of growth overfishing. The scarcity of large individuals implies that large and more important fish for reproduction are being removed from the stock. In the aspect of rebuilding the stock, management methods should prioritize the protection of large individuals and fish below, or near, maturity length in addition to regular monitoring, gear selectivity measurements, and independent sampling. These concerns will enhance spawning biomass and achieve the long-term sustainability of this crucial inland fishery resource for Egypt.

## Figures and Tables

**Table 1:** Monthly sampling frequency of *O. niloticus* individuals from Lake Nasser.

Month	Jan	Feb	Mar	Apr	May	Jun	Jul	Aug	Sep	Oct	Dec
Frequency	11	59	57	60	36	54	60	67	71	44	51

**Table 2:** The three growth model equations used in MMI model *.

Model	Equation	References
von Bertalanfy growth function (VBGF)	${L_{t}=L_{0}+\left(L_{\infty }-L_{0}\right)(1-e}^{-kt})$ (8)	[34]
Gompertz function	Lt=L0(elog(L∞L0)(1 − e−ga)) (9)	[35]
logistic function	$L_{t}=\frac{L_{\infty }L_{0}e^{gt}}{L_{\infty }\;+\;L_{0}e^{gt}\;-\;1}$ (10)	[36]

* As L_t_ is the length at age t, L_∞_ is the asymptotic length, and L_0_ is the length at birth. Each model has a unique growth coefficient (k = von Bertalanffy, g = Gompertz, and g = logistic). Although the Gompertz and logistic models use the same notation, the growth coefficients are not equivalent. However, the interpretation of L_∞_ and L_0_ is consistent across models.

**Table 3:** Fulton’s condition factor for male, female, and unidentified individuals of *O. niloticus*, Lake Nasser.

Sex	n	Mean (±SE)	SD	Min	Max
Female	225	2.06 ± 0.016	0.246	1.63	4.2
Male	283	2.05 ± 0.011	0.199	1.26	2.99

**Table 4:** The age agreement of readers for *O. niloticus*, Lake Nasser.

Fish Number	Readers	Agreement %	ASD *	ACV %	APE %
562	3	92.88	0.041	1.85	1.42

* ASD: average standard deviation.

**Table 5:** Length and age at maturity for *O. niloticus*, Lake Nasser.

Parameter	L_50_	L_95_	A_50_	A_95_
Male	29.93 ± 0.601	47.07 ± 1.761	2.45 ± 0.092	4.68 ± 0.277
Female	27.85 ± 1.047	46.80 ± 3.495	2.30 ± 0.069	4.96 ± 0.261
Total	29.41 ± 0.407	45.27 ± 1.163	2.47 ± 0.064	4.55 ± 0.194

**Table 6:** Multi-model inference (MMI) results for growth models for *O. niloticus*, Lake Nasser *.

Model	AICc	*Δ*	Weight (%)	RSE
VB	**5700.62**	0.00	0.64	**38.41**
Log	5704.98	4.35	0.07	38.56
Gom	5702.27	1.64	0.28	38.47

* The highlighted value in bold indicates the best model based on AIC and RSE.

**Table 7:** Bayesian inference growth parameters for *O. niloticus*, Lake Nasser *.

Parameters	Estimate	SD	Low-95% CI	Upper-95% CI	R^
Linf (cm)	47.71	1.08	45.57	49.70	1
K (year^−1^)	0.50	0.02	0.46	0.54	1
Sigma (cm)	4	0.35	3.7	4.91	1

* SD: the estimated error, and Sigma: the standard deviation of the observation.

**Table 8:** Key length parameters of *O. niloticus* with 95% confidence intervals (CI) *.

Parameters	Mean_Estimate	Lower CI	Upper CI
L_max_	49.98	47.99	51.94
L∞	52.61	50.51	54.68
L_mat_	29.32	27.15	31.59
L_opt_	30.80	27.65	34.20
L_opt_p10_	33.88	30.41	37.63
L_opt_m10_	27.72	24.88	30.78

* All lengths measured in cm.

**Table 9:** Froese’s length-based indicators of *O. niloticus* with 95% confidence intervals (CI).

Parameters	Froese Catch (Mean)	Lower CI	Upper CI	Froese Target
1 P_mat_	44.75	32.00	61.09	100
2 P_opt_	35.56	23.11	46.53	100
3 P_mega_	18.14	4.82	37.94	20

**Table 10:** Growth parameters and key length parameters of *O. niloticus* at different regions reported by previous authors *.

Location	Growth Parameters			Length-Based Indicators			
	L∞	K	b	L_max_	L_mat_	L_opt_	Author
Lake Edku	31.08 TL 27.50 TL	0.31 0.52		29.53 26.13	21.7 19.8	24.3 22.4	Soliman (2005) [42] El-Sawy (2006) [43]
Rosetta branch, River Nile	28.50 TL	0.39		27.08	20.4	23	Mahmoud and Mazrouh (2008) [44]
Tropical shallow lake in Mexico	17.88 TL	0.34		16.99	13.0	15.5	Gómez-Márquez et al. (2008) [45]
Lake Burullos	34.62 TL	0.21		32.89	23.7	26.4	Sangak (2010) [46]
Nozha Hydrome	38.06 TL	0.21	2.91	36.16	21.9	24.5	Mahmoud et al. (2013) [47]
Lake Naivasha, Kenya	25.4 TL (males) 25.9 TL (females)			24.13 24.61	17.7 18	20.2 20.5	Otieno et al. (2014) [48]
Amerti reservoir	31.6 TL (males) 27.3 TL (females)			30.02 25.94	21.5 18.9	24.1 21.5	Hailu (2014) [49]
Lake Manzala	34.51 TL		3.08	32.79	20.2	22.9	Mehanna et al. (2020) [3]
Koka Reservoir, Ethiopia	35.6 TL	0.37		33.82	24.4	27.1	Jemal and Aemro (2022) [50]
The Kafue Flood Plain	27.4 TL			26.03	18.98	21.6	Mbewe et al. (2023) [51]
Lake Nasser	40.8 TL (males) 40 TL (females)			38.76 38	27.5 27	30.3 29.8	AbouelFadl et al. (2024) [52]
Roseries Reservoir, Sudan	45.15 TL	0.31	2.80	42.89	29.7	32.4	Shuaib et al. (2025) [53]
Samendéni reservoir, Burkina Faso	33.6 TL	0.44	3.1	31.92	22.9	25.6	Ouédraogo et al. (2025) [54]
Lake Nasser	52.61 TL	0.50	3	49.98	29.32	30.80	Present study (2021)

* TL: Total body length, all lengths measured in cm, and K measured in year^−1^.

## Data Availability

The raw data supporting reported results will be available by the authors on request.

## Supplementary Materials

The following supporting information can be downloaded at https://www.mdpi.com/article/10.3390/biology15110868/s1, Table S1. Maturity stages description of *O. niloticus*, Lake Nasser; Table S2. Condition factor values (mean, minimum, and maximum) for males and females of *O. niloticus*, Lake Nasser across all collected months; Figure S1. The Gelman–Rubin diagnostic (R^) for growth parameters of *O. niloticus*, Lake Nasser; Figure S2. The posterior predictive check that compares observed data (dark blue line) to predicted values (light blue lines) based on the fitted model; Figure S3. The box plot of length parameters for *O. niloticus* shows the mean, upper, and lower confidence intervals.

## References

[1] FAO (2024). The State of World Fisheries and Aquaculture 2024.

[2] GAFRD (2022). Fish Statistics Yearbook.

[3] Mehanna S.F., Desouky M.G., Makky A.F. (2020). Growth, Mortality, Recruitment and Fishery Regulation of the Nile Tilapia *Oreochromis niloticus* (Teleostei: Cichlidae) from Manzala Lake, Egypt. Iran. J. Ichthyol..

[4] Saber M.A., Aly W. (2023). Size Selectivity of Trammel Nets Applied in Small-Scale Fisheries of Lake Nasser, Egypt. Egypt. J. Aquat. Res..

[5] WorldFish (2018). Management Plan for the Lake Nasser Fishery: Stock Assessment Study.

[6] Zambrano L., Martínez-Meyer E., Menezes N., Peterson A.T. (2006). Invasive Potential of Common Carp (*Cyprinus carpio*) and Nile Tilapia (*Oreochromis niloticus*) in American Freshwater Systems. Can. J. Fish. Aquat. Sci..

[7] Absar A., Chadha N.K., Joshi K.D., Chakraborty S.K., Sawant P.B., Kumar T., Sharma A.P. (2015). Maturation Profile and Fecundity of the Exotic *Oreochromis niloticus* in the River Yamuna, India. J. Environ. Biol..

[8] Tripathi S., Gopesh A., Dwivedi A.C. (2017). Framework and Sustainable Audit for the Assessing of the Ganga River Ecosystem Health at Allahabad, India. Asian J. Environ. Sci..

[9] Dwivedi A.C., Tiwari A., Mayank P. (2018). Environmental Pollution Supports the Constancy and Invader Potential of *Cyprinus carpio* and *Oreochromis niloticus* from the Ganga River, India. Int. J. Poult. Fish. Sci..

[10] Beaune D., Guillard J., Cottet M., Kue K., Lae R., Chanudet V., Descloux S., Tessier A. (2020). Investigating Key Biological Parameters of Nile Tilapia (*Oreochromis niloticus* L.) in a Large Asian Reservoir to Better Develop Sustainable Fisheries. Hydroecol. Appl..

[11] Halls A., Habib O.A., Nasr-Allah A., Dickson M. (2015). Lake Nasser Fisheries: Literature Review and Situation Analysis.

[12] Froese R. (2004). Keep It Simple: Three Indicators to Deal with Overfishing. Fish. Fish..

[13] Hordyk A.R., Loneragan N.R., Prince J.D. (2015). An Evaluation of an Iterative Harvest Strategy for Data-Poor Fisheries Using the Length-Based Spawning Potential Ratio Assessment Methodology. Fish. Res..

[14] Mildenberger T.K., Taylor M.H., Wolff M. (2017). TropFishR: An R Package for Fisheries Analysis with Length-Frequency Data. Methods Ecol. Evol..

[15] Froese R., Winker H., Coro G., Demirel N., Tsikliras A.C., Dimarchopoulou D., Scarcella G., Probst W.N., Dureuil M., Pauly D. (2018). A New Approach for Estimating Stock Status from Length Frequency Data. ICES J. Mar. Sci..

[16] Abdalla M.Y.M., Shuaib M.E.K., Alnaiem O., Hamid A.M., Adam A.E.B. (2024). Population Dynamics of Nile Tilapia (*Oreochromis niloticus*, Linnaeus. 1758) from Khashm El-Girba Reservoir, Atbara River, Eastern Sudan. Asian J. Fish. Aquat. Res..

[17] Ali A., Sarker M.R., Alam M.S. (2025). Development of a simple R package (aLBI) for the estimation of stock status from the length frequency data. Fish. Res..

[18] El-Haweet A., Adam E.-H., Sangq Y., Elfar A. (2008). Assessment of Lake Nasser Fisheries. Egypt. J. Aquat. Res..

[19] Silva M.I., Martins R., Sequeira V., Silva D., Farias I., Assis C.A., Gordo L.S., Vieira A.R. (2024). Struggling with Fish Age, a Comparison of Otolith Preparation Techniques to Unravel Age and Growth of Boarfish, *Capros aper* (Linnaeus, 1758). Sci. Rep..

[20] United Nations Environment Programme (UNEP) (2006). Africa’s Lakes: Atlas of Our Changing Environment.

[21] Abdellatif M., Mohammed-AbdAllah E., AbouelFadl K.Y., Osman A.G.M. (2022). Age and Growth of Chrysichthys Auratus (Geoffroy 1809) (Family: Claroteidae) from Lake Nasser, Egypt. Egypt. J. Aquat. Res..

[22] YEAG (Youth Employment in Aswan Governorate Project) (2017). Report of Fisheries Assessment of Lake Nasser, Aswan, Egypt.

[23] El-Far A., Aly W., El-Haweet A.E.D., Nasr-Allah A., Karisa H. (2020). Fisheries Management Based on Gear Selectivity of a Tropical Reservoir, Lake Nasser, Egypt. Egypt. J. Aquat. Res..

[24] Le Cren E.D. (1951). The Length-Weight Relationship and Seasonal Cycle in the Gonad Weight and Condition in the Perch (*Perca fluviatilis*). J. Anim. Ecol..

[25] Froese R. (2006). Cube Law, Condition Factor and Weight–Length Relationships: History, Meta-Analysis and Recommendations. J. Appl. Ichthyol..

[26] Gunderson D.R. (1993). Surveys of Fisheries Resources.

[27] Smart J.J., Chin A., Tobin A.J., Simpfendorfer C.A. (2016). Multimodel Approaches in Shark and Ray Growth Studies: Strengths, Weaknesses and the Future. Fish Fish..

[28] R Core Team R: A Language and Environment for Statistical Computing. https://www.R-project.org/.

[29] Walker T.I., Hamlett W.C. (2005). Reproduction in Fisheries Science. Reproductive Biology and Phylogeny of Chondrichthyans: Sharks, Batoids, and Chimaeras.

[30] Knoblauch K. (2014). Psyphy: Functions for Analyzing Psychophysical Data in R. http://CRAN.R-project.org/package=psyphy.

[31] Abouelfadl K.Y., Aly W., Osman A.G. (2020). Ageing Nile tilapia (*Oreochromis niloticus*): A comparative study between scales and otoliths. Int. J. Aquat. Biol..

[32] Beamish R.J., Fournier D.A. (1981). A Method for Comparing the Precision of a Set of Age Determinations. Can. J. Fish. Aquat. Sci..

[33] Chang W.Y. (1982). A Statistical Method for Evaluating the Reproducibility of Age Determination. Can. J. Fish. Aquat. Sci..

[34] von Bertalanffy L. (1938). A Quantitative Theory of Organic Growth (Inquiries on Growth Laws II). Hum. Biol..

[35] Ricker W.E. (1975). Computation and Interpretation of Biological Statistics of Fish Populations.

[36] Ricker W.E., Hoar W.S., Randall D.J., Brett J.R. (1979). Growth Rates and Models. Bioenergetics and Growth.

[37] Akaike H. (1973). Information Theory and an Extension of the Maximum Likelihood Principle. Second International Symposium on Information Theory.

[38] Anderson D.R., Burnham K.P. (2002). Avoiding Pitfalls When Using Information-Theoretic Methods. J. Wildl. Manag..

[39] von Doll J. (2024). Bertalanffy Growth Model Fitting with brms. fishR Blog. https://fishr-core-team.github.io/fishR/blog/posts/2024-2-5_LVB_brms/.

[40] Froese R., Binohlan C. (2000). Mpirical Relationships to Estimate Asymptotic Length, Length at First Maturity and Length at Maximum Yield per Recruit in Fishes, with a Simple Method to Evaluate Length Frequency Data. J. Fish Biol..

[41] Efron B. (1992). Bootstrap Methods: Another Look at the Jackknife. Breakthroughs in Statistics.

[42] Soliman T.B.H. (2005). Efficiency and Selectivity of Fishing Gears and Methods in Lake Edku and Their Effects on the Stock of Fish Populations.

[43] El-Sawy W.M.T. (2006). Some Biological Aspects of Dominant Fish Populations in Lake Edku in Relation to Prevailing Environmental Conditions.

[44] Mahmoud H.H., Mazrouh M.M. (2008). Biology and Fisheries Management of Tilapia Species in Rosetta Branch of the Nile River, Egypt. Egypt. J. Aquat. Res..

[45] Gómez-Márquez J.L., Peña-Mendoza B., Salgado-Ugarte I.H., Luis Arredondo-Figueroa J. (2008). Age and Growth of the Tilapia, *Oreochromis niloticus* (Perciformes: Cichlidae) from a Tropical Shallow Lake in Mexico. Rev. Biolgia Trop..

[46] Sangak Y.K. (2010). Distribution and Abundance of Fishes with Special Reference to Tilapia Species in Lake Burullos.

[47] Mahmoud H.H., Ezzat A.A., El-Sayed Ali T., El Samman A. (2013). Fisheries Management of Cichlid Fishes in Nozha Hydrodrome, Alexandria, Egypt. Egypt. J. Aquat. Res..

[48] Otieno O.N., Kitaka N., Njiru J.M. (2014). Length-Weight Relationship, Condition Factor, Length at First Maturity and Sex Ratio of Nile Tilapia, Oreochromis Niloticus in Lake Naivasha, Kenya. Int. J. Fish. Aquat. Stud..

[49] Hailu M. (2014). Gillnet Selectivity and Length at Maturity of Nile Tilapia (*Oreochromis niloticus* L.) in a Tropical Reservoir (Amerti: Ethiopia). J. Agric. Sci. Technol..

[50] Jemal K., Aemro D. (2022). Age and Growth of Nile Tilapia, Oreochromis Niloticus (Linnaeus, 1758), From Koka Reservoir, Ethiopia. Asian Fish. Sci..

[51] Mbewe I., Khondowe P., Mudenda H.G. (2023). Size at Maturity and Fecundity of Oreochromis Niloticus and Mouth Brooding Tilapiines Indigenous to Kafue Flood Plain Fishery, Zambia. Am. Acad. Sci. Res. J. Eng..

[52] AbouelFadl K.Y., AbouDeaf E.A.A., Mohammed M.K.S., Osman A.G.M. (2024). A Comparison of the Reproductive Patterns of Oreochromis Niloticus from Two Distinct Locations: Wadi Halfa, Sudan, and Aswan, Egypt. Egypt. J. Aquat. Biol. Fish..

[53] Shuaib M.E.K., Alttagi Z.E.A.A., Hamid A.M., Abdalla M.Y.M. (2025). Population Dynamics of Nile Tilapia (Oreochromis Niloticus, Linnaeus, 1758) from Roseries Reservoir, Sudan. Asian J. Fish. Aquat. Res..

[54] Ouédraogo R.B., Sanogo S., Compaoré I. (2025). Population Dynamics of Nile Tilapia, Oreochromis Niloticus (Linnaeus, 1758) in Samendéni Reservoir, Burkina Faso. Fish. Aquat. Sci..

[55] Murua H., Rodriguez-Marin E., Neilson J.D., Farley J.H., Juan-Jordá M.J. (2017). Fast versus Slow Growing Tuna Species: Age, Growth, and Implications for Population Dynamics and Fisheries Management. Rev. Fish Biol. Fish..

[56] Bagenal T.B., Tesch F.W., Bagena T.B. (1978). Methods for Assessment of Fish Production in Fresh Waters.

[57] Wootton R.J. (1990). Ecology of Teleost Fish.

[58] Ragheb E. (2023). Length-Weight Relationship and Well-Being Factors of 33 Fish Species Caught by Gillnets from the Egyptian Mediterranean Waters off Alexandria. Egypt. J. Aquat. Res..

[59] Blackwell B.G., Brown M.L., Willis D.W. (2000). Relative Weight (Wr) Status and Current Use in Fisheries Assessment and Management. Rev. Fish. Sci..

[60] Wootton R.J. (1998). Ecology of Teleost Fishes.

[61] Paugy D., Lévêque C., Teugels G.G. (2003). Poissons d’eaux Douces et Saumâtres de l’Afrique de l’Ouest.

[62] King M. (2007). Fisheries Biology, Assessment and Management.

[63] Nikolsky G.V. (1963). Ecology of Fishes.

[64] Nandikeswari R., Sambasivam M., Anandan V. (2014). Estimation of Fecundity and Gonadosomatic Index of Terapon Jarbua from Pondicherry Coast, India. Int. Sch. Sci. Res. Innov..

[65] Balogun A.T., Ajibare A.O., Ojo O.B. (2025). Sex Ratio, Gonado-Somatic Index and Hepato-Somatic Index of Coptodon Zilli and Oreochromis Niloticus Inhabiting Ureje Reservoir, Ado-Ekiti. Fudma J. Sci..

[66] Wagaw S., Sisay A., Bazezew A., Enawgaw Y., Wosnie A. (2024). Biological Aspects of Oreochromis Niloticus (Linnaeus, 1758) in Geray Reservoir (Ethiopia) for Effective Sustainable Fisheries. Fish. Aquat. Sci..

[67] Lowerre-Barbieri S.K., Ganias K., Saborido-Rey F., Murua H., Hunter J.R. (2011). Reproductive Timing in Marine Fishes: Variability, Temporal Scales, and Methods. Mar. Coast. Fish..

[68] Paloheimo J.E. (1980). Estimation of Mortality Rates in Fish Populations. Trans. Am. Fish. Soc..

[69] Catalano M.J., Allen M.S. (2010). A Size- and Age-Structured Model to Estimate Fish Recruitment, Growth, Mortality, and Gear Selectivity. Fish. Res..

[70] Vitale F., Worsøe Clausen L., Ní Chonchúir G. (2019). Handbook of Fish Age Estimation protocols and Validation Methods.

[71] Froese R., Pauly D. FishBase. https://www.fishbase.org.

[72] Hassan A., El-Kasheif M. (2013). Age, Growth and Mortality of the Cichlid Fish Oreochromis Niloticus (L.) from the River Nile at Beni Suef Governorate, Egypt. Egypt. J. Aquat. Biol. Fish..

[73] Grammer G.L., Slack W.T., Peterson M.S., Dugo M.A. (2012). Nile Tilapia Oreochromis Niloticus (Linnaeus, 1758) Establishment in Temperate Mississippi, USA: Multi-Year Survival Confirmed by Otolith Ages. Aquat. Invasions.

[74] El-Kasheif M.A., Authman M.M.N., Al-Ghamdi F.A., Ibrahim S.A., El-Far A.M. (2015). Biological Aspects and Fisheries Management of Tilapia Fish Oreochromis Niloticus (Linnaeus, 1758) in El-Bahr El-Faraouny Canal, al-Minufiya Province, Egypt. J. Fish. Aquat. Sci..

[75] Berihun A., Mingist M., Abebe G., Aemro D., Mequanent D. Age Determination and Growth of Nile Tilapia, *Oreochromis niloticus*, in Lake Hayq, Ethiopia. Proceedings of the World Aquaculture Safari.

[76] Burnham K.P., Anderson D.R. (2002). Model Selection and Multimodel Inference: A Practical Information-Theoretic Approach.

[77] Sparre P., Venema S.C. (1998). Introduction to Tropical Fish Stock Assessment. FAO Fish. Tech. Pap..

[78] Gelman A., Shalizi C.R. (2013). Philosophy and the Practice of Bayesian Statistics. Br. J. Math. Stat. Psychol..

[79] Prince J.D., Hordyk A.R. (2019). What to Do When You Have Almost Nothing: A Simple Quantitative Prescription for Managing Extremely Data-Poor Fisheries. Fish Fish..

[80] Panda D., Mohanty S.K., Pattnaik A.K., Das S., Karna S.K. (2018). Growth, Mortality and Stock Status of Mullets (Mugilidae) in Chilika Lake, India. Lakes Reserv..

[81] Roth A.D., Pilling S. (2005). Using an Evidence-Based Methodology to Identify the Competencies Required to Deliver Effective Cognitive and Behavioural Therapy for Depression and Anxiety Disorders. Behav. Cogn. Psychother..

