# Supplementary material for: Bayesian Growth Modeling and Length-Based Indicators: Stock Assessment of Nile Tilapia (Oreochromis niloticus) in Lake Nasser, Egypt
Source: Biology (Basel). 2026 May 31;15(11):868. doi: 10.3390/biology15110868 (PMC13255802; doi:10.3390/biology15110868)
Supplement: Supplementary file 1 [file biology-15-00868-s001.zip › biology-4302000-supplementary.pdf]

Supplementary materials:

Table S1. Maturity stages description of *O. niloticus*, Lake Nasser.

Maturity stage	Description
Thread	Sex cannot be identified at this development level, and the gonads are threadlike.
Stage I (Immature or inactive)	The gonads are translucent, sex can be determined as male or female, and testes are smaller and thinner than the ovaries.
Stage II (resting)	The gonads are translucent and slightly bigger. The ovary's weight has increased. The testes do not contain sperm, but the ovaries do contain a few tiny eggs.
Stage III (Active or developing)	The gonads get larger, the volume and weight of the ovaries rise, and the testes become opaque in the absence of sperm. Ovaries are translucent and contain tiny eggs.
Stage IV (Active-ripe or developed)	The ovaries grow more, covering nearly the whole-body cavity. Testes are white, and when cut, a few sperm are discharged from the core. The ovaries have fully developed eggs.
Stage V (Spawning or Ripe-running)	Gonads are significantly larger and fill the entire body cavity. Milt and eggs are ejected by giving gentle pressure to the abdomen. The ovary is currently claimed to be in the running phase.
Stage VI (Spent)	The ovaries are flaccid, with a few weakened eggs within them, and the testes are nearly empty. Some unspawned huge ova and an abnormally large number of tiny ova are present.

Table S2. Condition factor values (mean, minimum, and maximum) for males and females of *O. niloticus*, Lake Nasser across all collected months.

Month	Sex	n	Mean ± SE	Minimum	Maximum
January	Female	3	0.77 ± 0.42	0.34	1.62
	Male	8	0.08 ± 0.03	0.01	0.24
February	Female	30	0.61 ± 0.16	0.03	3.10
	Male	25	0.11 ± 0.01	0.02	0.42
March	Female	24	0.57 ± 0.18	0.04	3.10
	Male	28	0.13 ± 0.03	0.01	0.48
April	Female	36	**0.89 ± 0.17**	0.03	3.67
	Male	20	**0.27 ± 0.06**	0.02	0.73
May	Female	16	0.58 ± 0.20	0.05	2.56
	Male	17	0.07 ± 0.01	0.02	0.23
June	Female	15	0.39 ± 0.18	0.05	2.50
	Male	32	0.05 ± 0.01	0.01	0.41
July	Female	20	0.23 ± 0.1	0.00	1.87
	Male	27	0.07 ± 0.00	0.00	0.29
August	Female	20	0.44 ± 0.1	0.00	2.32
	Male	32	0.13 ± 0.04	0.01	0.98
September	Female	23	0.43 ± 0.52	0.02	2.32
	Male	45	0.16 ± 0.1	0.00	1.90
October	Female	14	0.25 ± 0.1	0.1	1.21
	Male	27	0.1 ± 0.04	0.01	0.79
December	Female	24	0.33 ± 0.12	0.023	2.69
	Male	22	0.1 ± 0.3	0.01	0.71

*The highlighted value in bold indicates the GSI peak for females (April) and for males (May).

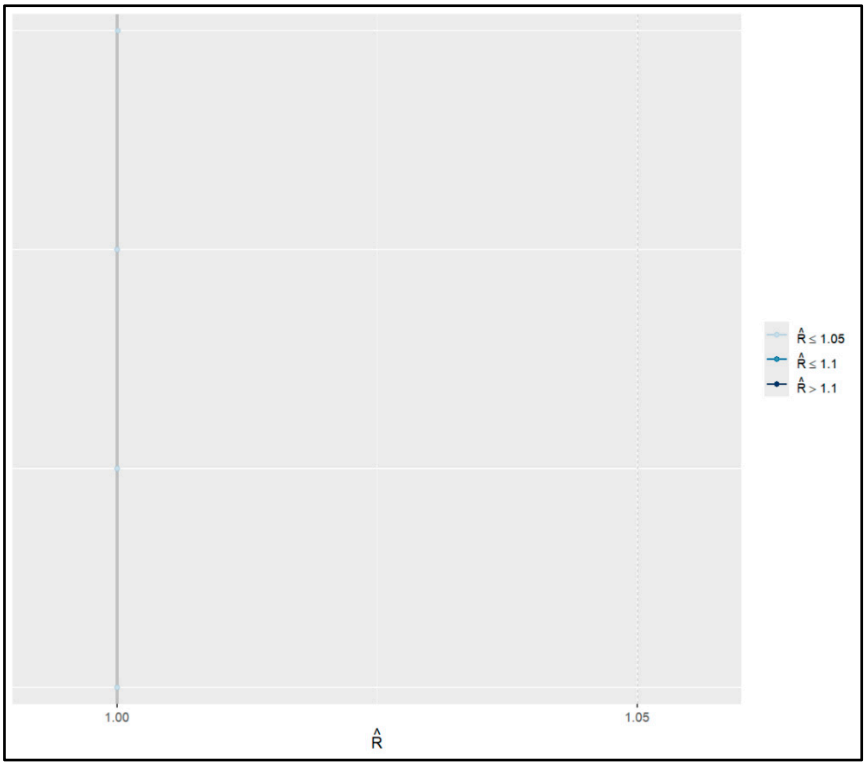

Figure S1. The Gelman-Rubin diagnostic (R^) for growth parameters of *O. niloticus*, Lake Nasser.

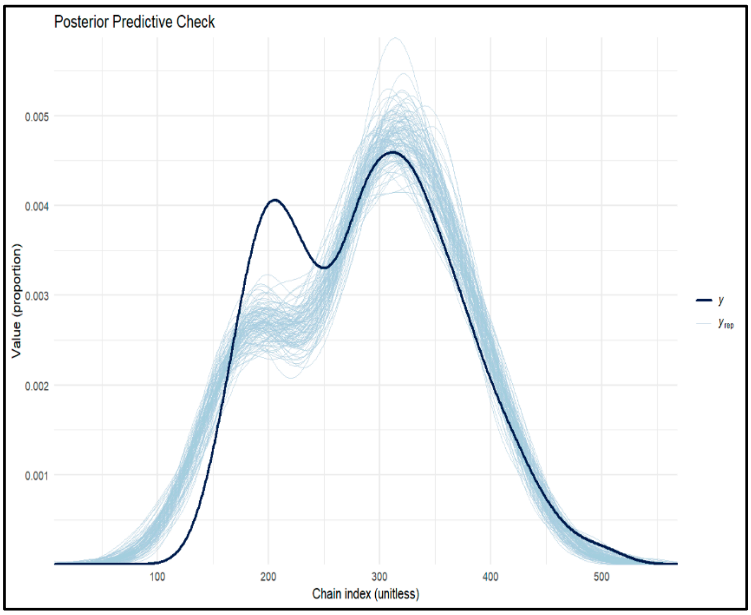

Figure S2. The posterior predictive check that compares observed data (dark blue line) to predicted values (light blue lines) based on the fitted model.

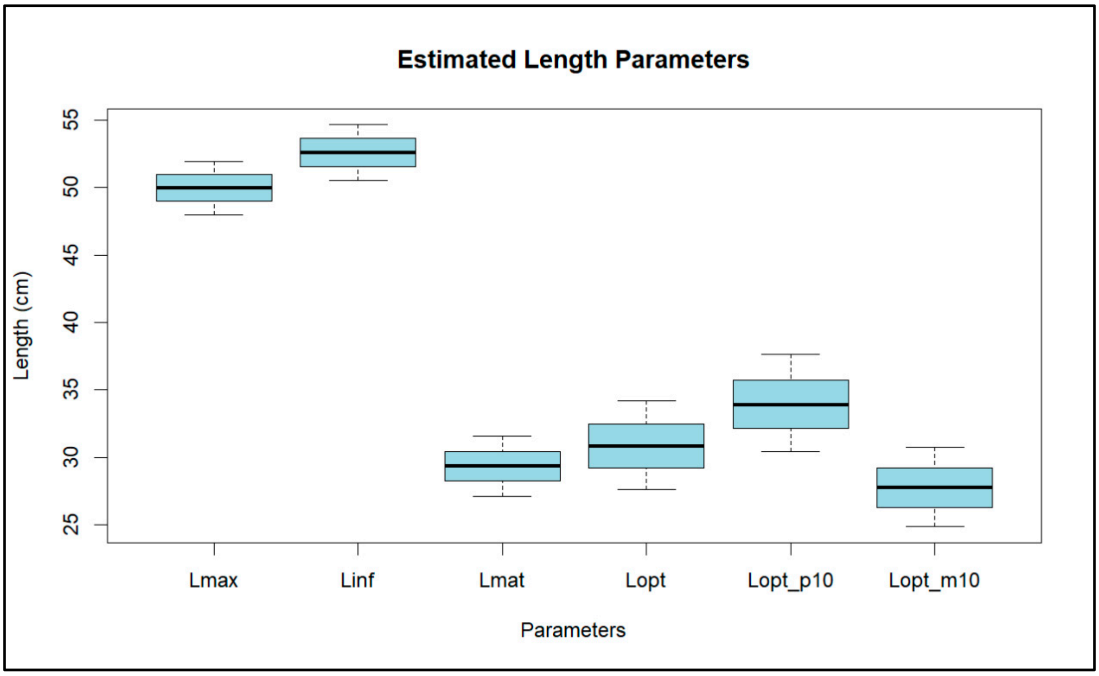

Figure S3. The box plot of length parameters for *O. niloticus* shows the mean, upper, and lower confidence intervals.
